# Bubble rising and interaction in ternary fluid flow: a phase field study

**DOI:** 10.1039/d2ra06144a

**Published:** 2023-01-25

**Authors:** Mingguang Shen, Ben Q. Li

**Affiliations:** a School of Mathematics and Statistics, Yancheng Teachers University Yancheng 224002 PR China; b Department of Mechanical Engineering, University of Michigan Dearborn MI 48128 USA benqli@umich.edu +1 (313)593-5241

## Abstract

Bubble–droplet interaction is essential in the gas-flotation technique employed in wastewater treatment. However, due to the limitations of experimental methods, the details of the fluid flow involved have not been fully understood. Therefore, a phase field model for a three-phase flow was developed to study the rise of a single bubble and bubble–droplet interactions. The fluid–fluid interfaces are tracked by the Cahn–Hilliard equation, which is coupled with the Navier–Stokes equations with an equivalent volumetric force substituted for interfacial tensions. The model was discretized using an explicit finite difference method on a half staggered grid, and the pressure velocity coupling was tackled using the projection method. The in-house code was written in Fortran and run with the help of OpenMP, a shared memory parallelism. The model was validated against experiments with gratifying agreement achieved. Bubble–droplet interaction was simulated in two distinct situations: the first features a gas bubble crossing the interface between two other phases, and the second features a gas bubble chasing from behind an oil droplet in a surrounding fluid of the third phase.

## Abstract 2

Bubble–droplet interaction is essential in the gas-flotation technique employed in wastewater treatment.

## Graphical abstract

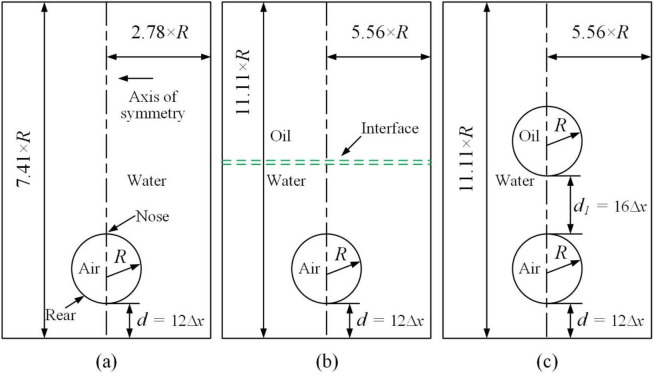

## 1. Introduction

Bubble rising, a type of multiphase flow phenomenon, occurs in many industrial applications, such as boiling,^1–3^ oil processing,^4–6^ and exploitation of combustible ices.^7–9^ A better understanding of bubble rising dynamics is thus crucial in developing optimal processing parameters for these processes. Previous studies, both numerical and experimental, have been mostly on the behavior of a single bubble rising in a heavier medium, and on the rising velocity, aspect ratio, terminal shape, and the like.^10–12^ In different flow conditions, denoted by a couple of dimensionless parameters, like the Eötvös number (defined later on), bubbles display distinct final shapes and rising velocities. Besides, some empirical correlations for the rising velocity have been proposed.^13–15^ The interactions of two or more bubbles of the same fluid have been investigated by Tripathi *et al.*,^16^ who found that the destabilizing nature of the wake leads to oscillatory trajectories.

Among numerical techniques to investigate bubble rising dynamics of a binary fluid system is the phase field method.^17–19^ Distinct from traditional sharp interface methods, it embraces a thin but finite interface region instead of a sharp one of vanishingly small thickness.^20^ In phase field modeling, the tracking of fluid–fluid interfaces *via* the Cahn–Hilliard equation is implicit, and its discretization and coding are easier to handle in comparison with other methods such as the volume of fluid method and the front tracking method. Moreover, the Cahn–Hilliard model boasts of sound conservation of mass, which is however a matter of concern in the level set method. Last but not least, with the nowadays GPU technology, the phase field method could be readily extended to three dimensions without the adaptive mesh refinement technique.^21^

The phase field model could be extended to more than three phases as well. Kim^22^ proposed a phase field model for ternary fluids, with the Cahn–Hilliard equation to track the interface between each pair of components. The interfacial tension is expressed as a volumetric term in the Navier–Stokes equations. This model conforms to the requirements of consistency and degeneracy.^23,24^ Consistency means that when a phase is absent at the beginning it will be so all the time. Degeneracy states that when a phase is absent a ternary model degenerates into a binary one. Kim^22^ did not consider contact line dynamics for ternary fluid flow. Nevertheless, the issue has been studied by a number of scholars.^25–28^ Other phase field models for ternary fluid flow can be found in a review paper.^29^

Phase field modeling of the interaction between two immiscible drops could be found in a few studies.^30–32^ They however mainly focused on the engulfing mechanism in a confined shear flow, and treated all the fluids as having equal densities and viscosities. For vertical head-on collisions of two immiscible droplets, Zhang *et al.*^33^ investigated the effect of the ratio of interfacial tensions on the film thickness, the maximal deformation, and the contact time. It is noticed that the two droplets that were vertically aligned crashed into each other in the process.

To the authors' best knowledge, only a couple of studies concerning the gas-flotation technique in wastewater treatment were conducted numerically, as by Ming *et al.*^34^*via* a smooth particle hydrodynamics model and by Kalantarpour *et al.*^35^*via* a phase field Lattice Boltzmann method. Kalantarpour *et al.*^35^ also simulated bubble–droplet interaction in water, but their model cannot access all the range of parameters, which is however not a matter of concern in the model by Kim.^22^

Having surveyed the literature and found that little effort is paid to understand the details of fluid flow in the gas-flotation technique, this paper is therefore dedicated to that end, with a view to gaining better understanding into the mechanism behind the gas-flotation technique in wastewater treatment. The paper is organized as follows. First, mathematical statement and numerical schemes are given, followed by the mesh convergence study to determine a reasonable spatial step. Second, effect of the phase field mobility was examined. Third, single bubble rising across the interface between two other immiscible fluids was simulated. Fourth, bubble–droplet interaction in a heavier medium was studied. The phase field mobility was found to drastically influence bubble rising velocity and was adjusted based on the experimental work by Liu *et al.*^36^

## 2. Mathematical statement

### 2.1 Mass and momentum equations

The governing equations for triple phase flows of incompressible Newtonian fluids turn up as follows, with eqn (1) being the continuity equation and eqn (2) the Navier–Stokes equation. *D***u**/*Dt* in eqn (2) is the material derivative. The third term on the right hand side of eqn (2) represents interfacial tension, which will be defined later on.

1 ∇·**u** = 0

2
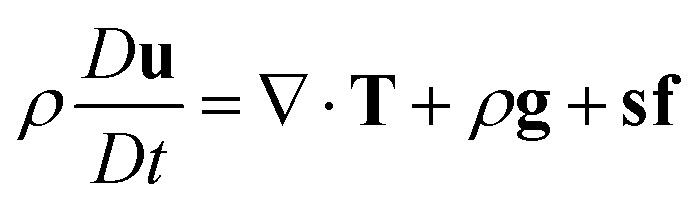


In the equations above, **u** is velocity, *t* is time and **T** = −*p***I** + **σ** is the total stress tensor. *p* is the mechanical pressure and **σ** = μ[∇**u** + (∇**u**)^*T*^] is the Newtonian stress tensor, with *μ* being viscosity. **g** stands for the local gravitational acceleration. It is to be noted that all of the field parameters, like *ρ* = *ρ*_1_*c*_1_ + *ρ*_2_*c*_2_ + *ρ*_3_*c*_3_, are to be defined as continuous functions of the order parameters. Besides, *c*_1_ + *c*_2_ + *c*_3_ = 1, where the subscripts represent different phases. *c*_1_ = 1 represents water, *c*_2_ = 1 air and *c*_3_ = 1 Oliver oil.

### 2.2 Phase field equation for an evolving fluid–fluid interface

In this paper, the motion of a fluid–fluid interface is described by the Cahn–Hilliard equation, which tracks the interface implicitly with the phase indicator *c*_*i*_.3
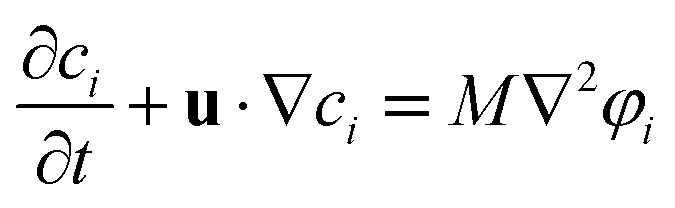
4
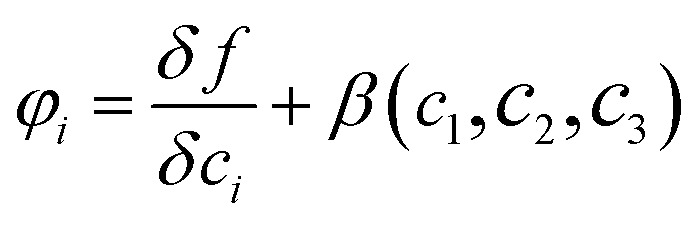
where *M* is the diffusion coefficient and *φ*_*i*_ is the chemical potential, with 
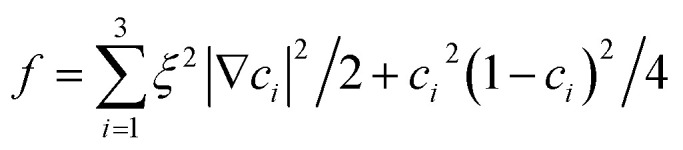
 being the bulk free energy density. *ξ* stands for a measure of interfacial thickness. *β*(*c*_1_,*c*_2_,*c*_3_) in eqn (4) is a Lagrange-multiplier to ensure 
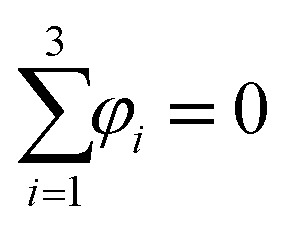
. Since *c*_1_ + *c*_2_ + *c*_3_ = 1, only two of them needs updating. Additionally, the diffusion strength *M* and the characteristic interface thickness *ξ* are assumed constant and equal for each fluid–fluid interface.

### 2.3 Interfacial tension

The interfacial tension between any two phases is converted to a volumetric force in phase field modeling. In this paper, a volumetric counterpart of capillary force according to Kim^22^ is adopted. It takes on the following form.5
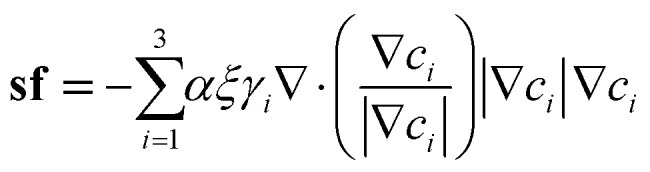
where *α* = 6√2 and *γ*_*i*_ is the phase specific interfacial tension coefficient, satisfying the relationship *σ*_*ij*_ = *γ*_*i*_ + *γ*_*j*_. *σ*_*ij*_ refers to the interfacial tension between phase *i* and phase *j*. Therefore, for triple phase systems, *γ*_*i*_ is uniquely determined.6
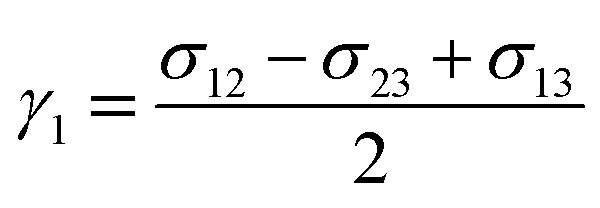
7
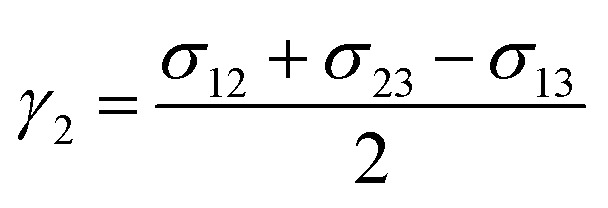
8
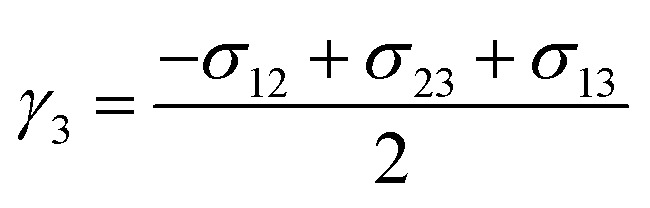


### 2.4 Boundary/initial conditions and numerical procedures

**Fig. 1:**
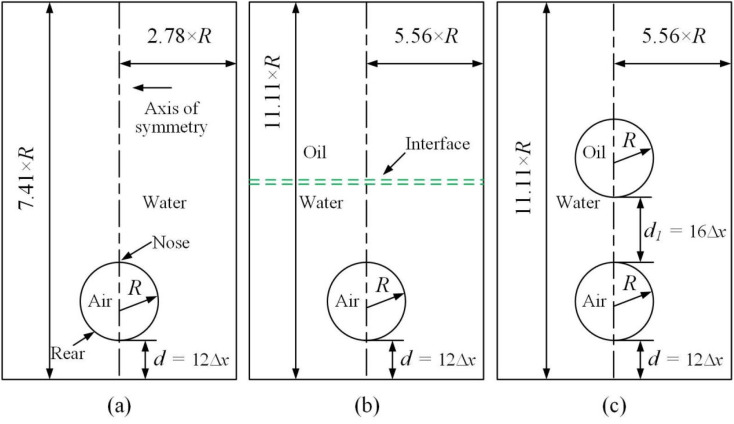
Schematic of the problem. (a) For Section 3.1 to 3.3, (b) for Section 3.4 and (c) for Section 3.5.

Boundary conditions are needed to close a set of partial differential equations. The schematic of the problem is given in Fig. 1, with all the borders being treated as walls. Initially, the air bubble sits about 12Δ*x* above the bottom in a quiescent fluid. The dashed line in Fig. 1(b) symbolizes an interface. The effect of computational domain has been ruled out. Besides, the diameters of the bubble and the droplet, unless otherwise stated, is set to 2.7 mm throughout the paper.

**Fig. 2:**
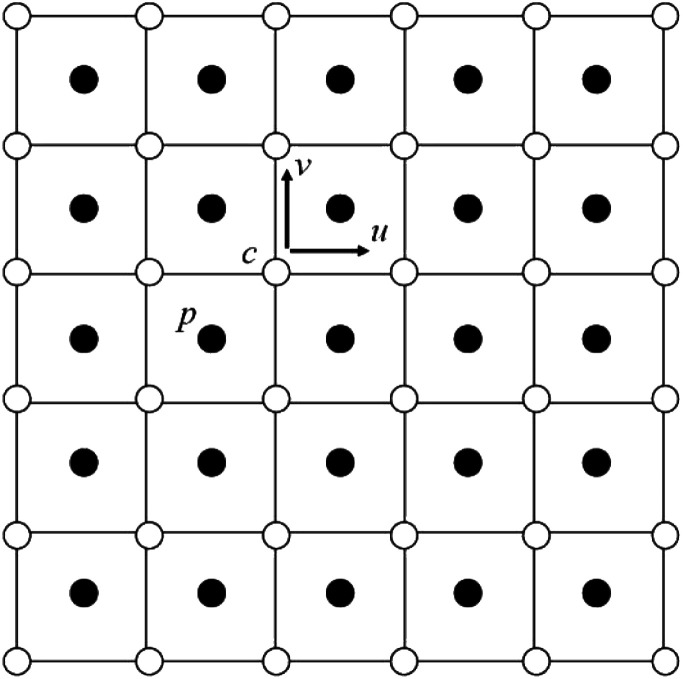
A half staggered grid. It is noted that only pressure is stored at the cell center, while all the others are stored at the cell vertices.

Thanks to the symmetric motion of bubble rising, only half computational domain enters calculation. Zero Neumann boundary conditions are applied on all the walls and the axis of symmetry for the chemical potential, the order parameters, and pressure. As for velocity, the normal component of velocity vanishes and the tangential component is mirrored on the axis of symmetry. The no slip condition is imposed on all the walls. An explicit finite difference method on a half-staggered grid is adopted, as shown in Fig. 2.

**Fig. 3:**
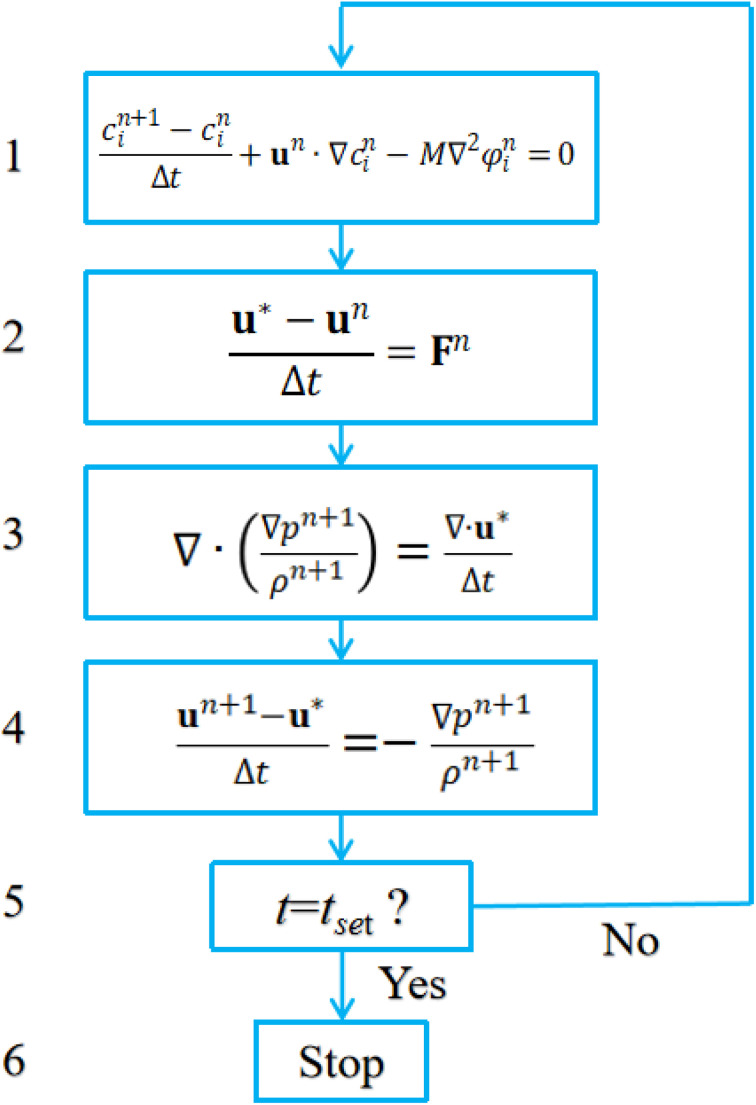
Flow chart of the algorithm.

As for the discretization schemes, traditional central difference schemes are employed to discretize diffusion terms, and upwind schemes to approximate convection terms. The discretization procedure of eqn (5) could be found in Kim.^22^ During a time marching step, computation starts from the evolution of *c*_*i*_, and then proceeds to the intermediate velocity u*, followed by the updating of the pressure *p*^*n*+1^ and by the renewal of the velocity **u**^*n*+1^, thus completing one marching. It will, however, not stop until the time duration set is reached. The solution step is summarized as follows in Fig. 3, where the superscript *n* flags the previous time step and *n* + 1 the current. **F**^*n*^ contains all the other terms in the momentum equation. The time step Δ*t* is on the order of magnitude of 10^−6^ s.

**Fig. 4:**
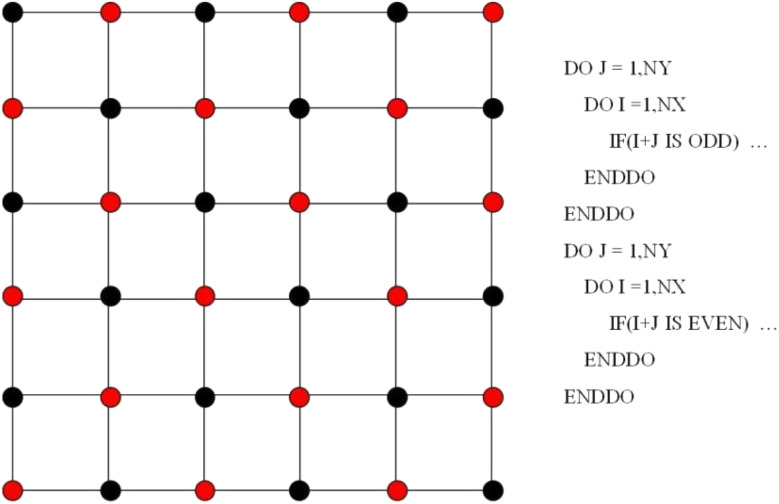
Checkboard partition for the Red/Black SOR algorithm of the pressure Poisson equation in step 3 in Fig. 3. Right is the pseudo Fortran code.

In addition, parallel programming based on OpenMP is utilized to accelerate computation. The pressure Poisson equation in step 3 in Fig. 3 is tackled using a Red/Black SOR algorithm, which is a parallel version of the traditional SOR algorithm. Fig. 4 describes a domain partition pattern of the Red/Black SOR algorithm. Notice that this partition pattern is used only for the updating of pressure.

As indicated in Fig. 4, their update is divided into two steps. When the first group (red, for instance) is renewed using the values only at the black points, the second group (black) is updated using the newest values only at the red points. In this way, data dependency could be eliminated. Data dependency happens when a processor is reading the value and another is modifying the value of the same point in the meantime. The processor reading the value may or may not get the newest value of that point depending on the capabilities of the two processors. This would however not happen in the chessboard ordering. Besides, in each step, the successive over relaxation (SOR) method could be employed.

## 3. Results and discussion

In this section, the mesh sensitivity study was conducted, followed by the validation of the model and by the investigation of the effect of the phase field mobility on bubble rising. Then, two distinct cases in triple phase flow were examined. The thermophysical quantities used are listed in Table 1, with those for Oliver oil found in Sahasrabudhe *et al.*^37^ The word and/or numeral in the parenthesis indicates that the surface energy is about the interface between the substance of interest and that in the parenthesis. Air is denoted as component 2, water component 1 and Oliver oil component 3.

**Table 1:** Thermophysical quantities used in this paper

	Oliver oil (3)	Water (1)	Air (2)
Density (kg m^−3^)	900	1000	1.18
Viscosity (mPa s)	75	1	0.0185
Surface energy (mN m^−1^)	32 (air)	72 (air)	
	16.82 (water)		

### 3.1 Mesh sensitivity study

For phase field models, mesh convergence study means two things: how many nodes to resolve the thin interface and how thin the interface thickness should be compared with certain macroscopic length scales, for instance, bubble diameter. The first has been solved with the characteristic interface thickness *ξ* set equal to the spatial step Δ*x*.^38^ The second is to be decided with the following mesh sensitivity study, where an air bubble rising in water was simulated on grids of varied resolutions. A dimensionless number Cn = *ξ*/*D*, defined as the ratio of the characteristic interface thickness *ξ* to the bubble diameter *D*, is also employed to indicate the fineness of the grid.

For convenience of discussion, a couple of dimensionless number are defined.^39^ They are listed as follows.9
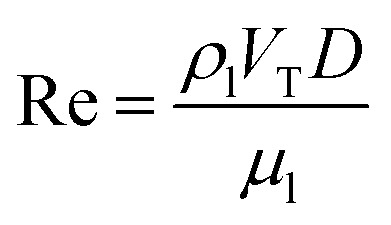
10
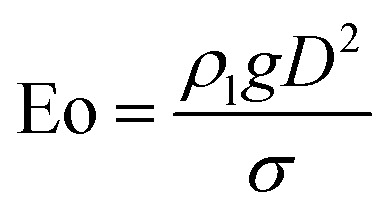
11
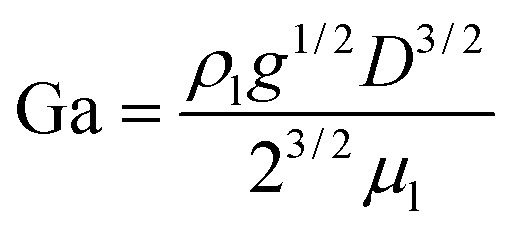


Eqn (9) defines the Reynolds (Re) number, measuring the relative importance between inertial and viscous forces, and eqn (10) defines the Eötvös (Eo) number, measuring the relative importance between gravitational and surface tension forces. The last is the Galilei (Ga) number. As the terminal velocity *V*_T_ is not a prior, the Re number is less used.

**Fig. 5:**
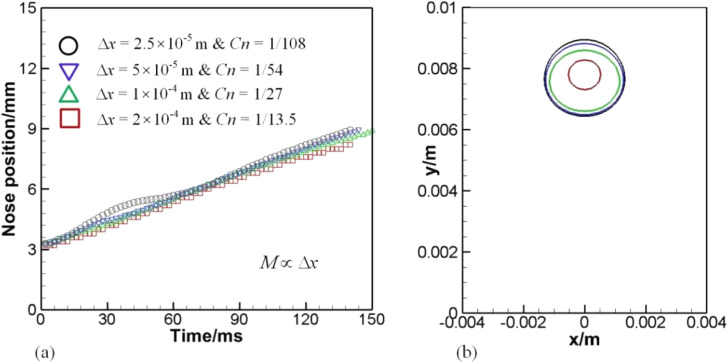
(a) Documents the history of the nose position and (b) depicts the bubble shape at 140 ms.

In the equations above, *ρ*_l_ denotes liquid density, *V*_T_ signals the terminal velocity of the bubble, *μ*_l_ stands for liquid viscosity, and *σ* surface tension coefficient. In this section, Re ∼ 108, Ga ∼ 155, and Eo ∼ 1 if the terminal velocity *V*_T_ is assumed to be on the order of magnitude of 0.04 m s^−1^. The numerical outcome is shown in Fig. 5, where for instance Cn = Δ*x*/(54Δ*x*) = 1/54 means there are 54 cells across the bubble diameter.

Fig. 5(a) plots the time evolution of the nose position of the bubble. Even with the coarsest grid, the captured position agrees quite well with the others. The relative error of the predicted top positions between the intermediate (Cn = 1/54) and finest grids (Cn = 1/108) at 140 ms is only 1.4%. The deviation in the finest grid around 40 ms is probably due to the stronger acceleration and to the choosing of the phase field mobility. As demonstrated in Section 3.2, the larger the phase field mobility, the higher the rising velocity. However, there is no consistent law to select the phase field mobility when performing the mesh refinement study for phase field modeling. Therefore, this is possibly caused by the fitting parameters.

Fig. 5(b) displays the bubble shape on various grids at 140 ms, where an evident loss of mass occurs if the coarsest grid is employed. As the mesh is refined, the problem is alleviated. Though the Cahn–Hilliard model can well conserve the total mass of a binary phase flow, the mass of one component may not be conserved, which has been also observed.^40,41^ The shrinkage of drops can be reduced with the Cn number set below a critical value, typically on the order of magnitude of *O*(10^−2^) as suggested by Yue *et al.*^40^

**Fig. 6:**
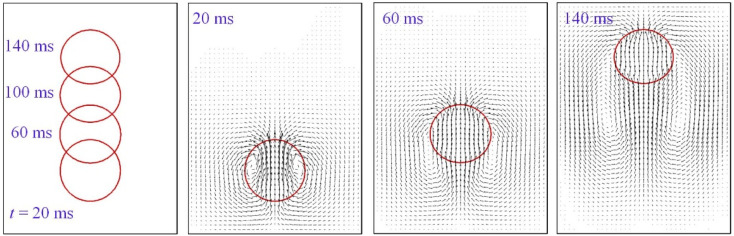
Bubble shape and velocity field at different instants on the grid of Δ*x* = 5 × 10^−5^ m.

Given the result of the mesh sensitivity study here, the grid with Δ*x* = 5 × 10^−5^ m or Cn = 1/54 is employed, unless otherwise stated, throughout the paper. Another issue is the choice of the phase field mobility, which has been adjusted according to the mesh size *M* ∼ 0.2Δ*x* to achieve the sharp interface limit. Fig. 6 shows a sequence of bubble shapes and velocity field for the gird of Δ*x* = 5 × 10^−5^ m.

The leftmost column in Fig. 6 traces the history of bubble shapes, and the rest columns give velocity field distribution in the computational domain. Though a liquid jet below the bubble is induced as shown at 20 ms, it is not strong enough to pierce into the bubble. Moreover, the bubble, although slightly elliptical as shown at *t* = 20 ms and 140 ms, is almost spherical during the rising process.

**Fig. 7:**
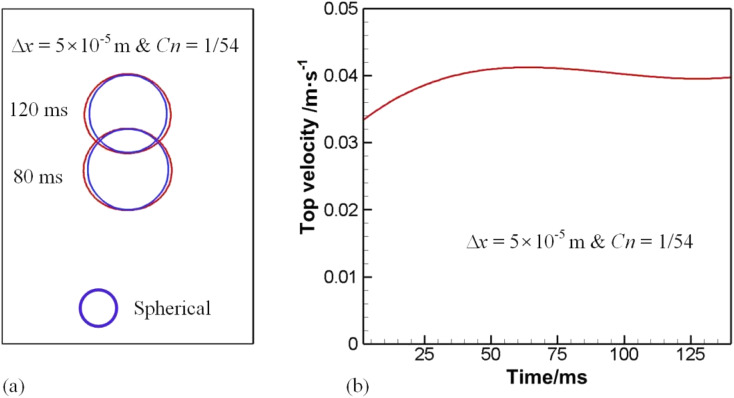
(a) Deviation of bubble shape from spherical to elliptical and (b) history of the bubble rising velocity.

The velocity of the top of the bubble is given in Fig. 7(b), which shows that it is nearly constant except at the very beginning when it experiences an acceleration. Chen *et al.*^10^ also noticed this.^10^ It is worth noting that the bubble is a prolate at *t* = 20 ms in Fig. 6, a shape that helps rise up, as was pointed out by Yang *et al.*^18^ who showed that a uniform vertical electric field elongates a bubble in the direction of rising, thereby speeding up the bubble. As the bubble passes the initial period of acceleration, the bubble shape appears to have been evolved as well. Inspection of Fig. 7(a) shows that the rising bubble, when reaching a steady state, becomes an oblate ellipsoid. This is consistent with the phase diagram proposed by Bhaga and Weber.^42^

### 3.2 Validation of the model

**Fig. 8:**
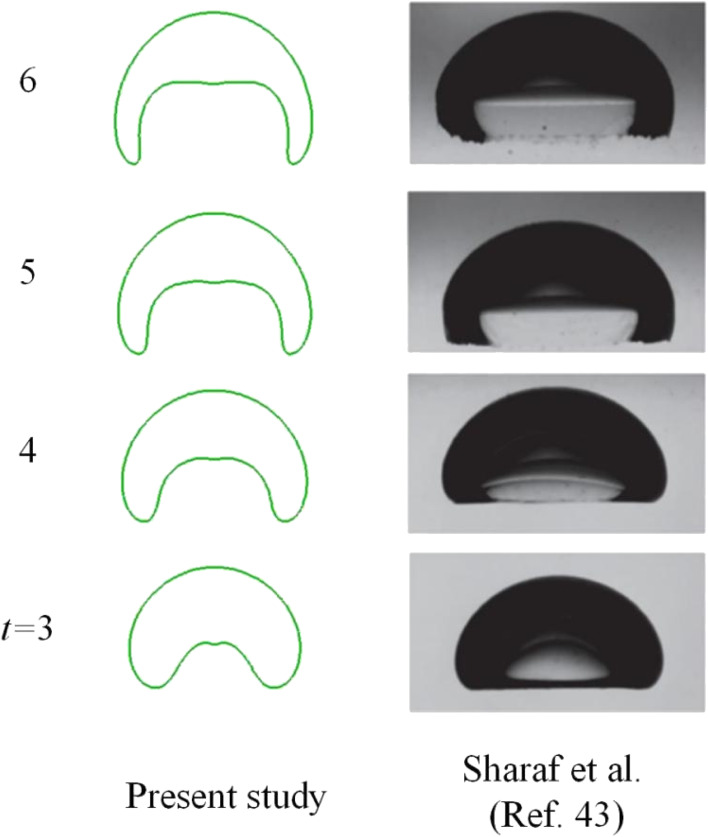
Comparison of bubble shape with experimental results by Sharaf *et al.*^43^

Having performed mesh sensitivity studies, attention is paid to the comparison of computed results with experimental data to validate the phase field model. In the case above, surface tension dominates as is evident by the Eötvös number. In this section, inertia dominated cases were considered, where the experiment conducted by Sharaf *et al.*^43^ was used for comparison. The drop radius is 19.27 mm and the numerical outcome is provided in Fig. 8. In this section, Ga ∼ 2960, and Eo ∼ 50, meaning strong inertia but weak surface tension effect.

Fig. 8 displays a bubble resembling a skirted cap at *t* = 6. Overall speaking, the comparison between numerical and experimental data is reasonable. As the bubble starts moving upwards due to buoyancy around *t* = 3, a vortex flow as in Fig. 6 will develop around the periphery, resulting in a large dynamic pressure on the bottom of the bubble. Since the dynamic pressure inside the bubble is much less than outside the bubble, the pressure difference dimples the bottom, as demonstrated in Fig. 8. The gas inside would be accelerated, rushing to the upper surface, where the pressure gradient is initially lower than on the bottom. As the bottom continues deforming, the gas inside pushes further the upper surface, rendering it to move.

**Fig. 9:**
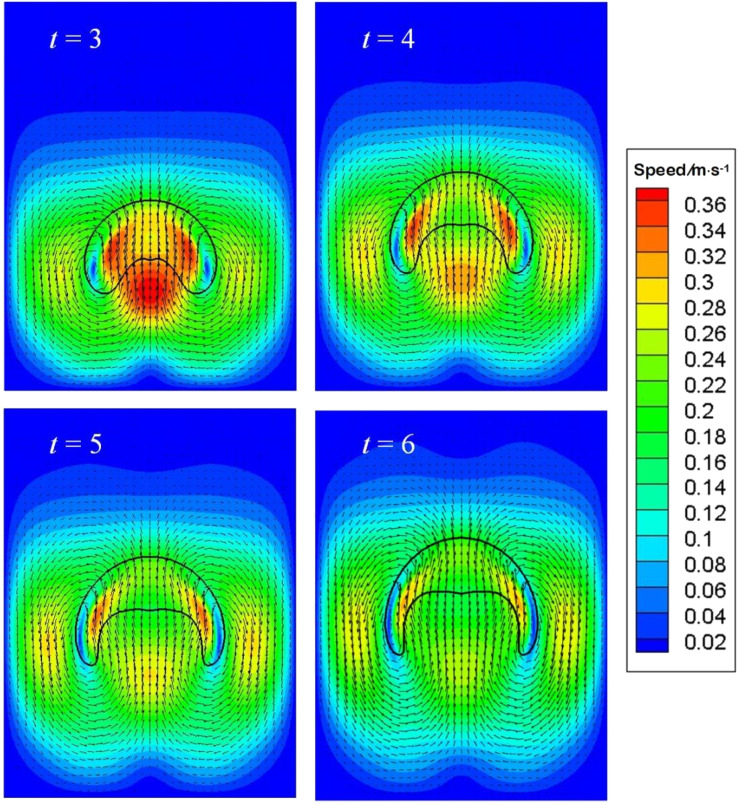
Speed distribution at different instants for the validation case.

Fig. 9 gives the speed distribution at particular instants. At *t* = 3 when the bottom has been deformed due to the liquid jet from the difference between buoyancy and drag resistance, it is clear that the vortex core comes near the skirt, facilitating its growing. The maximum speed shows up in the wake at *t* = 3. The uprising jet then brings the air in motion *via* its viscosity. As the jet pushed by pressure gradient continues forging ahead, the distance between the top and bottom however would cease increasing at the center line, because of a higher pressure developed in front of the jet, or more precisely beneath the top surface of the bubble. Thus a negative pressure gradient forms, reducing the velocity of the jet, as shown at *t* = 4. Meantime, the wake velocity also dwindles due to viscosity. Since the vortex core sits near the skirt, the skirt is elongated, which in turn thins the vortex pattern and leads to an expanded and flattened bottom, as displayed at *t* = 5 and *t* = 6.

### 3.3 Effect of the phase field mobility on an air bubble rising in water

In a phase field model, an important parameter is the phase field mobility. In general, it should not be too large lest it dampens convective motion, nor can it be too small since it can increase unwanted deformation. Not easily amenable to experimental observation, its choice is mostly empirical, often on the order of magnitude of *χ*Δ*x*^2^ with *χ* being a tuning parameter. In the current model, *M* ∼ *χ*Δ*x* is chosen due to the incorporation of *ξ*^−1^ into *M* and to the scale of *ξ* ∼ Δ*x*. Different phase field mobilities may lead to distinct fluid behaviors. Therefore, it is of practical importance to check its effect on bubble rising.

**Fig. 10:**
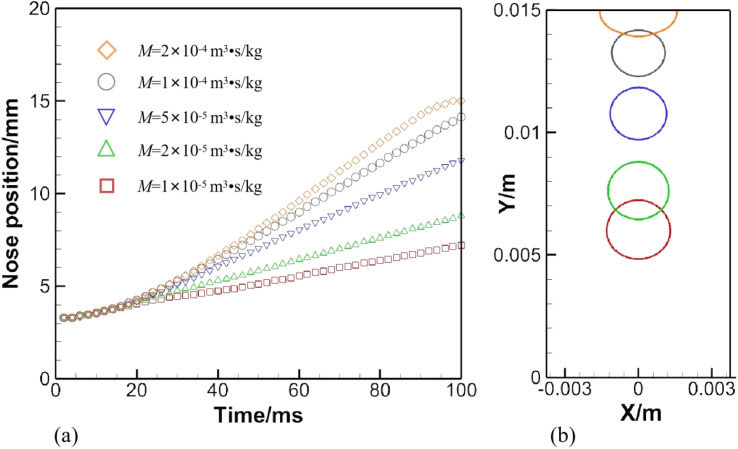
(a) Effect of the phase field mobility on an air bubble rising in water. (b) Depicts bubble shapes under various phase field mobilities at 100 ms.

A set of computed results is given in Fig. 10, where numerical configurations are the same as in Section 3.1, except for the phase field mobility, which is allowed to vary. The history of the nose position under a variety of phase field mobilities is provided in Fig. 10(a), and the bubble shape at 100 ms is shown in Fig. 10(b).

The effect of the phase field mobility on the rising speed is evident in Fig. 10(a), if not drastic. Basically, the rising velocity is roughly proportional to the phase field mobility. The little plateau formed by the orange diamond in Fig. 10(a) shows that the bubble has been in contact with the top awhile. Another matter of concern can be identified in Fig. 10(b), where a loss of mass is aggravated as the phase field mobility is increased. The problem can be alleviated through a reasonable choice of the phase field mobility and *via* a proper volume ratio between the bubble and the whole computational domain.

Though the mesh convergence study helps choose a proper spatial step, the terminal rising velocity has not been validated against existing experimental or theoretical data. Liu *et al.*^36^ conducted experiments on air bubbles rising in water, and graphed the rising velocity against the dimensionless number, Eo = *gD*^2^(*ρ*_l_ − *ρ*_g_)/*σ*. The definition here is slightly different from eqn (10), which neglects gas density. The rising velocity, derived from the nose position in Fig. 10(a) when *M* = 5 × 10^−5^ m^3^ s kg^−1^, approaches that predicted by Liu *et al.*,^36^ which is on the order of magnitude of *O*(10^−1^). Consequently, the phase field mobility is fixed to 5 × 10^−5^ m^3^ s kg^−1^ throughout the following sections.

### 3.4 An air bubble crossing the oil–water interface driven by buoyancy force

**Fig. 11:**
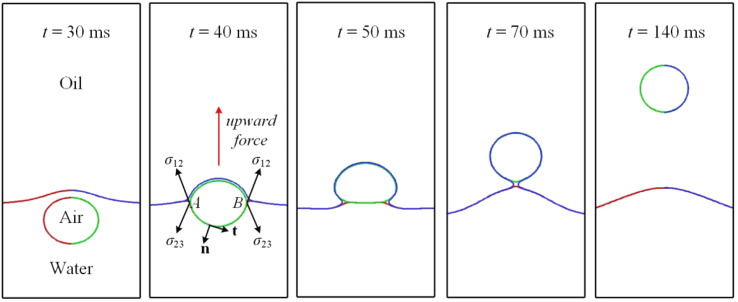
A sequence of snapshots showing an air bubble rising across the oil–water interface.

The foregoing sections deal only with binary phase flows, for which phase field models can be very useful. This section examines an air bubble rising in two different phases, with the lighter Oliver oil floating on the heavier water. Since the bubble may cross the interface between the other two phases, the effect of interfacial tensions may bring about some interesting results. Computational configuration is the same as in Fig. 1(b) and Fig. 11 depicts a sequence of snapshots showing an air bubble rising across the oil–water interface.

Fig. 11 is obtained by extracting the contours of *c*_1_ = *c*_2_ = *c*_3_ = 0.5 and by mirroring the right half with respect to the axis of symmetry. The air bubble at *t* = 30 ms is depicted intentionally in both green and red, since the bubble at this instant is completely submerged in water so that the contours of *c*_1_ = *c*_2_ = 0.5 coincide with each other. As the air bubble rises close to the oil–water interface, the flow in front of the bubble will deform the interface, driving it upwards. At *t* = 30 ms, the bubble appears to be spherical in shape, having an aspect ratio of around unity. As the bubble continues rising upwards, making its way through the oil–water interface around *t* = 40 ms, there is a net upward Laplace pressure driving it onwards, since the lower part of the bubble in contact with water has a larger interfacial tension than does the upper part in contact with oil. The bubble thus accelerates more in the rear.

This is worth elaborating upon. Integrating the surface tension force along the lower arc *AB* counterclockwise at *t* = 40 ms in Fig. 11, one has12
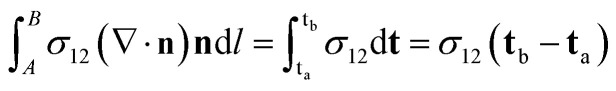


In eqn (12)**n** is the unit normal perpendicular to but pointing away from the arc *AB*, and **t** is the unit tangent normal. It is to be noted that **t**_a_ and **t**_b_ are collinear with *σ*_12_. Since surface tension *σ*_12_ makes an angle *θ*_1_ with the vertically upward *z* axis, the resultant lifting force of eqn (12) comes out as 2 × *σ*_12_ × cos *θ*_1_. In a similar manner, the effective drag force from surface tension *σ*_23_ would be 2 × *σ*_23_ × cos *θ*_2_. Given *σ*_12_ > *σ*_23_ and a comparable *θ*_1_ and *θ*_2_, one deduces that the combined force, pointing vertically upwards, would pull the bubble up. In addition, the bottom experiences stages of shape evolution, from semicircular in the water to flattened across the interface, and then to semicircular again if it is completely wetted by oil as shown at *t* = 140 ms in Fig. 11.

As the bubble gradually departs from the oil–water interface, the extra lifting force caused by the interfacial tension dwindles, since the interfacial tension gradually becomes uniform and the integral in eqn (12), with the kernel *σ*_23_d**t**, could be integrated along the whole periphery, meaning that it would vanish eventually. As a result, the bubble is subjected only to gravity, pressure, and viscous force. Completely submerged in oil, the bubble takes on again a spherical shape in accordance with the phase diagram proposed by Bhaga and Weber^42^ all the time from *t* = 70 ms to 140 ms.

Li *et al.*^44^ proposed four regimes for a bubble ascending in two media, with the lighter fluid being upon the heavier fluid. The results depend on the relative magnitude of interfacial tensions. The bubble could cross the interface between the two media if *σ*_12_ > *σ*_23_ + *σ*_13_ and *σ*_13_ < *σ*_23_ + *σ*_12_, which is satisfied in Fig. 11.

**Fig. 12:**
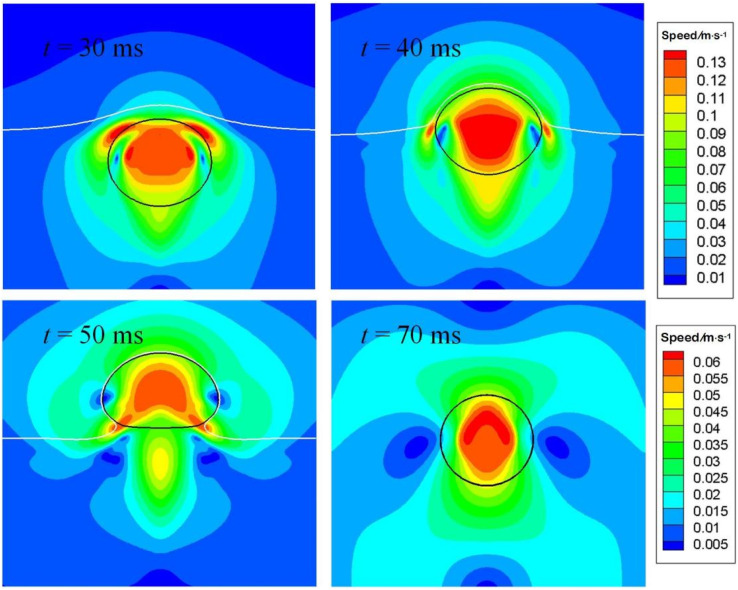
Flow fields around the air bubble as it is crossing the oil–water interface. The lower scale bar is only for *t* = 70 ms.

Fig. 12 depicts the distribution of speed for particular instants. At *t* = 30 ms, the bubble is making its way through the interface, the process of which resembles an impact on a soft substrate. Two lateral flows develop around the bubble nose, as shown therein due to mass conservation, with the speed being the largest. A pair of vortexes are also seen at *t* = 30 ms. As time progresses to 40 ms, the lateral flow moves downward, nearing the equator of the bubble. Meantime, the maximum speed within the bubble is located around the center, since the air velocity must be larger than the rising velocity of the bubble, so that the stagnation pressure beneath the top surface is higher than on it, generating a positive pressure gradient to drive the bubble to move. As the bubble is about to leave the interface as displayed at *t* = 50 ms, a cusp in the speed contour within the bubble is found, which is caused the extra acceleration induced by surface tension, as explained above.

**Fig. 13:**
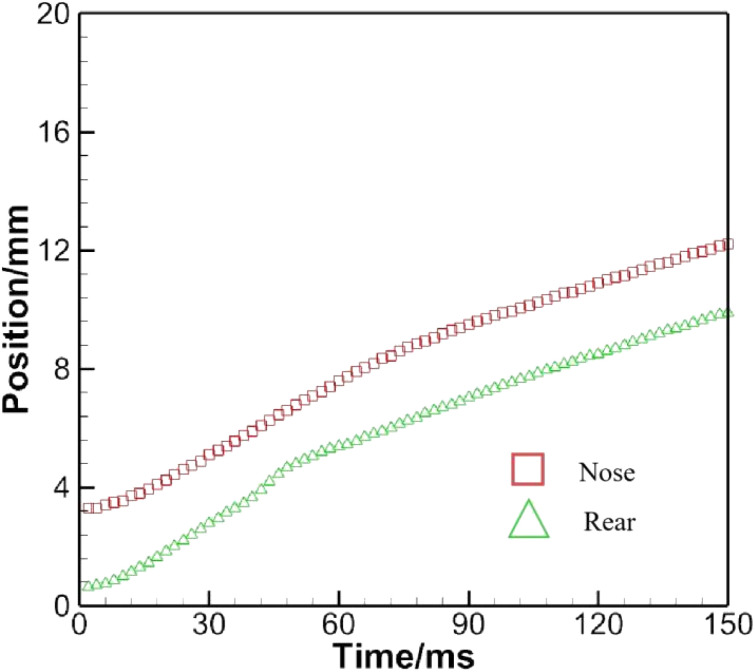
History of the positions of the nose and rear as a bubble crosses the oil–water interface.

Fig. 13 monitors the positions of the nose and the rear. These two lines are generally parallel to each other. Nevertheless, from *t* = 40 ms on, the distance between the nose and rear starts to decrease, reaching a minimum around *t* = 50 ms. Afterwards, it begins to increase until *t* = 70 ms and later remains essentially constant.

### 3.5 An air bubble rising behind an oil droplet in water

**Fig. 14:**
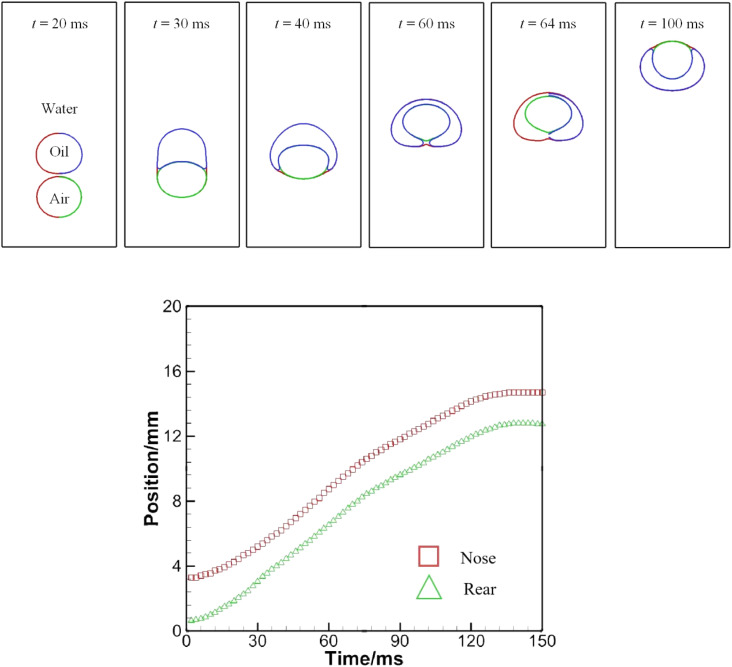
(a) A sequence of snapshots showing bubble–droplet interaction in water (upper). (b) History of the positions of the nose and rear as a bubble rising behind an oil droplet (lower).

A pair of bubbles or a combination of a bubble and a droplet, both of different fluids, interact with each other when they encounter during the process of rising up. There may be a number of possibilities when several bubbles rise and interact. If one from behind is moving faster than the other, it may push up the slower-moving one without direct contact, or may penetrate into the bigger one and then emerge out from the opposite side. Here the rising and interaction of an air bubble and an oil droplet in water is considered. Numerical configurations could be found in Fig. 1(c). Computed results of bubble–droplet interactions are plotted in Fig. 14.

Fig. 14 presents one possible outcome of bubble–droplet interaction. Initially, the air bubble is below the oil droplet. The air bubble strives to catch up with the slower oil droplet by squeezing the water in its front. At *t* = 30 ms, the bubble and the oil droplet have been in contact with each other and the oil droplet is deformed by the rising air bubble. Since the water–air interfacial tension is larger than the oil–air one, a resultant extra force helps further deform the oil droplet or help the bubble squeeze into the oil droplet, as explained at *t* = 30 ms in Fig. 11. At *t* = 40 ms, the air bubble appears to be surrounded only partially, but at *t* = 64 ms is swallowed up completely by the oil droplet. Subsequently, the air bubble, while being contained in the oil droplet, continues to ascend and attempts to separate from it because of a higher rising velocity due to buoyancy.

**Fig. 15:**
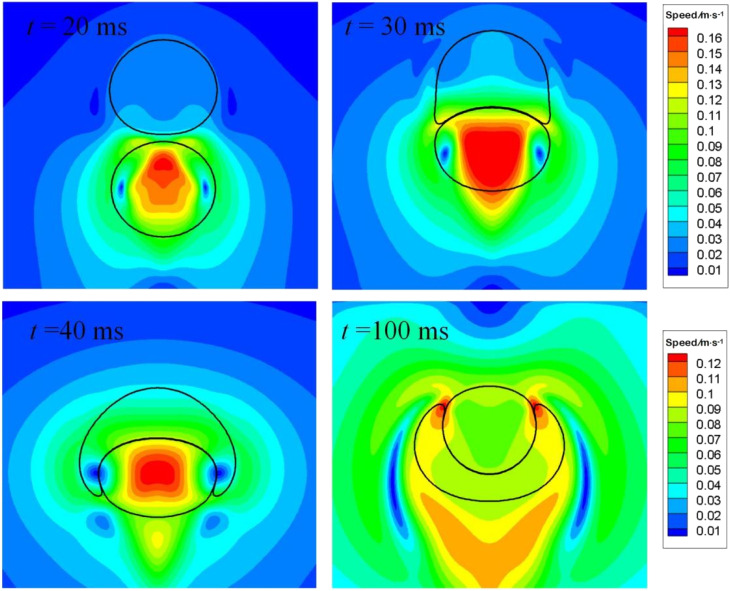
Speed distribution for the bubble–droplet interaction at particular instants. The lower scale bar is only for *t* = 100 ms.

The spreading coefficient, defined as *S* = *σ*_12_ − (*σ*_23_ + *σ*_13_), determines whether the droplet can spread on the bubble or whether the bubble can be wetted by the droplet. The spreading would occur if *S* is positive, which is the case at *t* = 64 ms as shown in Fig. 14(a). Inspection of the results in Fig. 14(b) shows that the distance between the nose and rear of the air bubble is reduced when the air bubble is to be swallowed up by the oil droplet, because the lower part of the air bubble is subjected to a higher interfacial tension, hence a larger acceleration rate. Afterwards, the distance changes little since an equilibrium state has been established. A similar case was studied by Kalantarpour *et al.*^35^ using a three-component phase field Lattice Boltzmann method. However, their model has some limitations: for instance, physical quantities like air viscosity are not assigned real values, but tailored for numerical convenience. Fig. 15 depicts the speed distribution for particular instants in Fig. 14. It is to be noted that the lower scale bar is only for *t* = 100 ms.

Fig. 15 indicates that when the bubble and the drop are to come in touch with each other, a higher pressure region would develop beneath the drop bottom to form a pressure gradient, driving the drop to rise. As they come in real touch, the bubble bottom would be deformed, resulting in an oblate. A large portion of air inside the bubble is also experiencing an thrust. As the bubble is being swallowed up by the drop, two pairs of vortexes develop as shown at *t* = 40 ms. As time proceeds to *t* = 100 ms, an equilibrium state established.

**Fig. 16:**
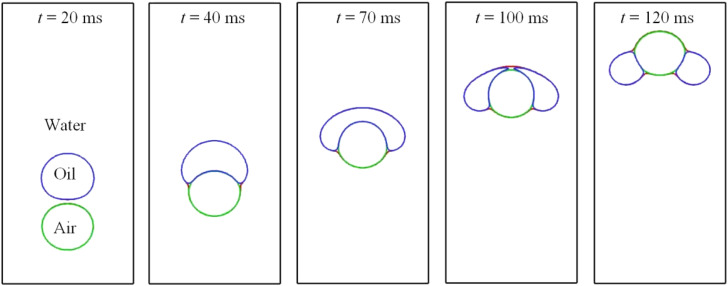
Bubble–droplet interaction when the air–water and air–oil interfacial tensions are the same.

Computed results with the air–water and the air–oil interfacial tensions both set to 0.072 N m^−1^ are plotted in Fig. 16. In this case, the air bubble experiences a uniform interfacial tension as it makes its way through the water–oil mixture. It is apparent from the figure that having dimpled the bottom of the oil droplet at *t* = 40 ms, the air bubble continues ascending, piercing into the oil droplet from below, and yet the droplet is unable to enclose the air bubble. Clearly, this is because the interfacial tension between air and oil is the same as between air and water herein. At *t* = 120 ms, the oil droplet evolves into a toroidal shape, signaling that the bubble is about to separate from the bubble. Besides, the spreading coefficient *S* herein is negative, indicating that full wetting of the bubble by the droplet is impossible, as shown in Fig. 16. It is worth noting that for the above two cases in Fig. 14 and 16, the rising of oil droplets is being accelerated by the air bubble, a process that can be of benefit to remove oil droplets in wastewater treatment.^45^

**Fig. 17:**
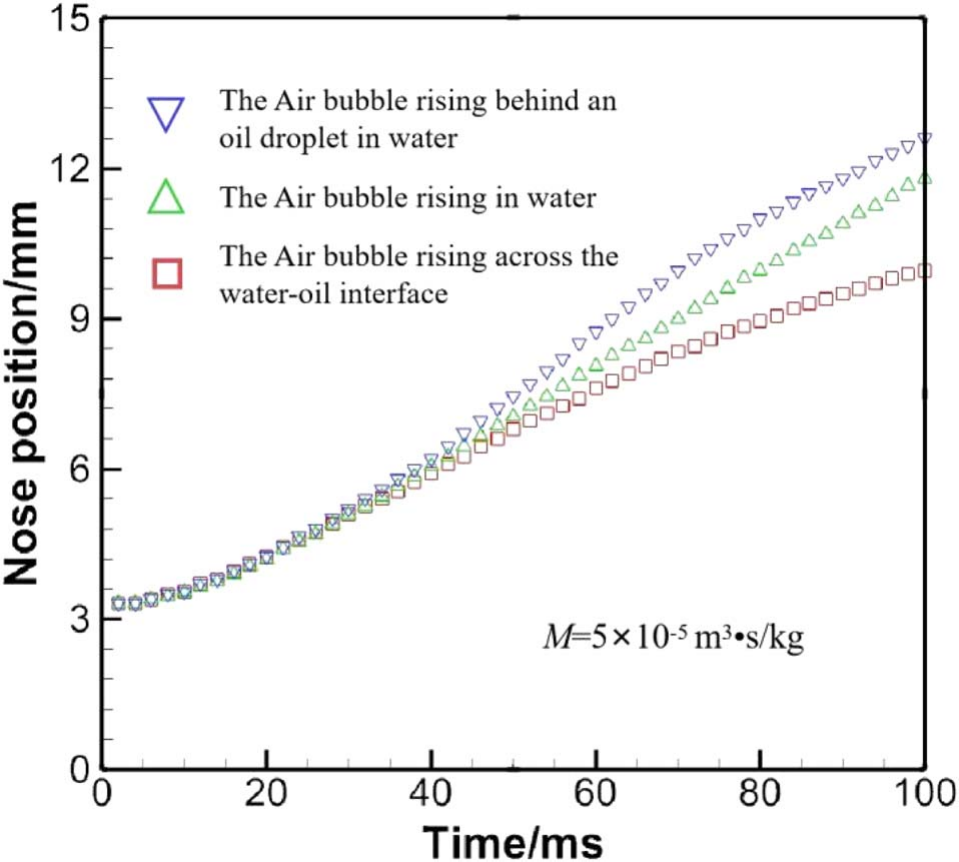
Comparison of the nose position of the air bubble under different circumstances.

Fig. 17 compares the nose positions of the air bubble under different conditions, where the air bubble is released at the same position. In general, the air bubble moves fastest when it is behind the oil droplet, and slowest when it is to cross water–oil interface, since the bubble becomes an oblate when crossing the interface, a geometric shape that hinders rising as verified by Yang *et al.*^18^

## 4. Concluding remarks

In this paper, a phase field model for ternary fluid flow was employed to examine bubble rising dynamics and bubble–droplet interaction in the gas-flotation technique. Three phase fields are updated each time step to track fluid–fluid interfaces. In single bubble rising dynamics, the phase field mobility was found to drastically influence the rising velocity. In the triple phase flow, the results indicate that by integrating the interfacial tension along the bubble perimeter, one would find an effective lifting force, pushing the bubble upwards as it tries to cross the oil–water interface. This resultant force could cause additional deformation on the bottom, inducing another clockwise vortex but retarding the upward motion. Besides, when chasing an oil droplet in water, the air bubble could merge with the oil droplet, floating together upwards at a faster pace. Though the 2D simulation could shed some light on the inner physics, the real situation is 3D. Therefore, further work could be done on fully 3D simulations in terms of interactions among a swarm of bubbles and droplets in a wide range of parameters, which may be made possible by the emerging GPU technology and may resemble more the factual situation in the gas-flotation technique.

## Author contributions

Conceptualization, M. S.; methodology, M. S.; software, M. S.; validation, M. S.; formal analysis, M. S.; investigation, M. S.; resources, M. S.; data curation, M. S.; writing—original draft preparation, M. S.; writing—review and editing, B. Q. L.; visualization, M. S.; supervision, B. Q. L.; project administration, B. Q. L.; funding acquisition, M. S. All authors have read and agreed to the published version of the manuscript.

## Conflicts of interest

None declared.

## Supplementary Material
